# Using Virtual Emergency Medicine Clinicians as a Health System Entry Point (Virtual First): Cross-Sectional Survey Study

**DOI:** 10.2196/42840

**Published:** 2023-08-03

**Authors:** Jennifer Potter, Dana Watson Gans, Alison Gardner, James O'Neill, Christopher Watkins, Iltifat Husain

**Affiliations:** 1 Department of Emergency Medicine Wake Forest University School of Medicine Winston-Salem, NC United States; 2 Department of Pediatrics University of Alabama Birmingham Birmingham, AL United States; 3 Novant Health Winston-Salem, NC United States

**Keywords:** telehealth, virtual care, emergency medicine, telemedicine, emergency department, acute care facilities, virtual visit, COVID-19, virtual, utilization, medicine, acute illness, illness, injury, patient, efficacy, infection, care, physician

## Abstract

**Background:**

The COVID-19 pandemic accelerated the use and acceptance of telemedicine. Simultaneously, emergency departments (EDs) have experienced increased ED boarding. With this acceptance of telemedicine and the weighty increase in patient boarding, we proposed the innovative Virtual First (VF) program to leverage emergency medicine clinicians’ (EMCs) ability to triage patients. VF seeks to reduce unnecessary ED visits by connecting patients with EMCs prior to seeking in-person care rather than using traditional ED referral systems.

**Objective:**

The goal of this study is to investigate how patients’ access to EMCs from home via the establishment of VF changed how patients sought care for acute care needs.

**Methods:**

VF is a synchronous virtual video visit at a tertiary care academic hospital. VF was staffed by EMCs and enabled full management of patient complaints or, if necessary, referral to the appropriate level of care. Patients self-selected this service as an alternative to seeking in-person care at a primary care provider, urgent care center, or ED. A postvisit convenience sample survey was collected through a phone SMS text message or email to VF users. This is a cross-sectional survey study. The primary outcome measure is based on responses to the question “How would you have sought care if a VF visit was not available to you?” Secondary outcome measures describe valued aspects and criticisms. Results were analyzed using descriptive statistics.

**Results:**

There were 3097 patients seen via VF from July 2021 through May 2022. A total of 176 (5.7%) patients completed the survey. Of these, 87 (49.4%) would have sought care at urgent care centers if VF had not been available. There were 28 (15.9%) patients, 26 (14.8%) patients, and 1 (0.6%) patient that would have sought care at primary care providers, EDs, or other locations, respectively. Interestingly, 34 (19.3%) patients would not have sought care. The most valued aspect of VF was receiving care in the comfort of the home (n=137, 77.8%). For suggested improvements, 58 (33%) patients most commonly included “Nothing” as free text.

**Conclusions:**

VF has the potential to restructure how patients seek medical care by connecting EMCs with patients prior to ED arrival. Without the option of VF, 64.2% (113/177) of patients would have sought care at an acute care facility. VF’s innovative employment of EMCs allows for acute care needs to be treated virtually if feasible. If not, EMCs understand the local resources to better direct patients to the appropriate site. This has the potential to substantially decrease patient costs because patients are given the appropriate destination for in-person care, reducing the likelihood of the need for transfer and multiple ED visits.

## Introduction

The COVID-19 pandemic accelerated patient use and acceptance of telemedicine platforms. Multiple studies across different subspecialties have demonstrated patient acceptance of and satisfaction with telemedicine visits [1-4]. A survey of 7477 patients with type 1 diabetes in 89 countries found that 86% of respondents saw utility in virtual appointments and 75% planned to use telemedicine appointments again [5].

Simultaneously, emergency departments (EDs) across the country have seen substantial fluctuations in volumes related to different variants of COVID-19. A retrospective observational study revealed that rates of boarding and access block had a statically significant increase during the COVID-19 pandemic when compared to control prepandemic periods [6]. During the early stages of the COVID-19 pandemic, total ED visits dropped by 42%-50% when compared to the same period of time in 2019 [6,7]. ED volumes in 2021 and 2022 have continued to be less than in 2019 by 10% and 12%, respectively [8]. The volume of low-acuity ED visits notably decreased, while the decrease in the volume of high-acuity patients was relatively small [6]. While the volume decreased, the resources available became increasingly limited, especially inpatient bed availability, which resulted in boarding. The effects of overcrowding and prolonged ED stays are not without consequences. Several studies have shown that ED length of stay has a significant negative effect on patient morbidity and mortality [9-11].

Traditionally, ED referrals come from primary care providers (PCPs), telephone consultation and triage services, urgent care centers (UCCs), and self-referrals. The ED does not take an active role in this referral process other than, at times, receiving notifications that patients are arriving. These referrals, as well as self-triage, can result in unnecessary visits to the ED. A study of the Healthdirect telephone referral system in Australia found that 10.3%, 26.2%, and 27.1% of patients were inappropriately triaged to the ED by general practitioners, patients themselves, and the Healthdirect telephone service, respectively [12]. Additionally, a study of 56 UCCs in Nevada found that 35.9% of UCC-to-ED referrals were unnecessary [13]. These unnecessary visits not only are costly to the patient but also contribute to the growing problem of ED overcrowding and boarding. Due to significant boarding, wait times, and the acceptance of telemedicine care during COVID-19, we proposed the innovative emergency medicine–led Virtual First (VF) program to leverage the emergency medicine clinicians’ (EMCs) ability to triage patients with acute illness. Currently, there is a paucity of data on using EMCs for virtual acute illness visits. There is, however, evidence to suggest that EMCs have the ability to effectively reduce unnecessary ambulance transports to the ED via telemedicine prior to patient arrival [14]. Moreover, there is growing literature regarding the efficiency [15,16], cost savings [17], and quality of care [15,18] provided by EMCs via telemedicine once the patient arrives in the ED. VF was marketed to patients as a platform to use before physically coming to the ED. Our VF program was staffed by EMCs and enabled patients to receive full management of their complaint or, if the EMC felt necessary, referral to a PCP, UCC, or ED. Patient complaints that VF would likely be able to divert from seeking additional in-person care include mild infectious complaints such as upper respiratory illness, diarrhea, vomiting, urinary tract infections, as well as minor trauma.

The goal of this study is to investigate how patients’ access to EMCs from home via the establishment of VF changed how patients sought care for acute care needs. We hypothesized that patients would seek care virtually for complaints that they would have otherwise sought care for within an ED.

## Methods

### Ethics Approval

Institutional review board approval (IRB00075547) was obtained after human subject ethical review and waiver of the requirements for signed informed consent was granted at our institution.

### Study Design

This is a cross-sectional survey. We performed an analysis of a prospectively collected postvisit survey sent to all patients that used the newly offered VF care option. VF, a synchronous virtual video visit for acute illness and injury, was started in April 2021 and staffed by EMCs, who were physicians, physician assistants, or nurse practitioners. The EMCs available via VF were emergency medicine physicians, physician assistants, and nurse practitioners. On the start date, patients were given the option of using VF when they accessed the MyChart app, a personal health access portal, or hospital website seeking emergency care. A MyChart account and video-capable computer or cell phone were required to successfully complete a VF visit. Surveys were collected during the first year of this service being made available to the public.

### Study Setting

VF is stationed at a tertiary care academic hospital and level one adult and pediatric trauma center with over 110,000 patient visits yearly. VF visits were conducted online through Epic MyChart. EMCs were available to patients via VF 24 hours a day, 7 days a week.

### Participants

A convenience sample of study participants included anyone that completed a VF visit for themselves or as guardians of children younger than 18 years. To participate in this study, the patient must have completed a VF visit. To complete a VF visit, a patient must be physically located within the state of North Carolina and have access to a smartphone or desktop computer. Patients with psychiatric complaints were not granted access to a VF visit. EMCs were instructed to send a postvisit survey to every patient seen. Since there were no patient identifiers, the results of the survey could not be linked to EMCs’ performance to avoid the bias of selecting only patients with positive experiences to receive a postvisit survey. There were no exclusion criteria for receipt of the postvisit survey. The patient proxy, as listed in the electronic medical record, was the selected recipient of the postvisit survey for patients younger than 18 years. Patients accessing care via VF more than once during the study period received a postvisit survey with each visit.

### Postvisit Survey

A postvisit survey link was sent by EMCs to patients who used our VF care option via Epic. Patients received this link through phone SMS text message or email. The postvisit survey was a nine-question unvalidated survey. Five questions were used to collect demographic information. The remaining questions were multiple-choice questions, with a free-text option, regarding previous use of our hospital system, patient self-triage if VF were not available, valued aspects of VF, and criticisms of VF. The questions regarding valued aspects and criticisms of VF allowed the patient to select multiple answers.

### Statistics

Survey answers were automatically entered into REDCap, a secure web-based survey building and data collection platform. Free-text responses to survey questions were individually reviewed by the authors and grouped into response categories. Some free-text responses fell into the originally defined categories from the survey question. For example, when asked about valued aspects of VF, one survey responder wrote “was very hard to move so not having to move made a huge difference.” This response was placed in the “comfort of home” category. Percentages of specific responses were calculated after analyzing the free-text responses.

## Results

There were 3097 patients seen via VF from July 2021 through May 2022. A total of 176 (5.7%) patients completed the postvisit survey. Of those patients, 164 (93.2%) were adults and 130 (73.9%) were women. Based on data from the statewide 2020 census, the races and ethnicities of survey respondents closely mimicked the racial and ethnic composition of the state at large. Of the 176 survey respondents, 150 (86.2%) were White, 20 (11.5%) were Black or African American, 2 (1.1%) were Asian, and 2 (1.1%) were Native American or Native Alaskan. Full demographic details are provided in Table 1.

VF primarily attracted patients that had previously been seen within the hospital system (n=169, 95.5%). As Table 2 shows, 7 (4%) survey respondents were new to the study site’s health care system.

If VF had not been available, 87 of 176 (49.4%) patients would have sought care at a UCC. As Table 3 shows, 28 (15.9%) patients, 26 (14.8%) patients, and 1 (0.6%) patient would have sought care at a PCP, ED, or other location, respectively. A total of 34 (19.3%) patients would not have sought care if VF had not been available.

Table 4 highlights the valued aspects of VF as well as the critiques. The most valued aspect of VF was the ability to receive care in the comfort of the home (n=137, 77.8%). Other valued aspects of VF included availability of appointment times (n=105, 59.6%), not having to wait in a lobby (n=100, 56.8%), and decreased infectious exposure (n=89, 50.6%). Valued aspects that were added as free text included instant access to care (n=5, 2.8%) and quality interaction with the EMC (n=4, 2.8%). One (0.6%) patient of 176 total patients responded that they did not value the appointment. For suggested improvements to VF, 58 (33%) patients included “Nothing” as free text, 47 (26.7%) suggested connectivity improvements, 23 (13.1%) wanted the ability to have lab work or imaging ordered as part of the visit, 14 (8%) did not like having to seek medical care after the VF visit, and 10 (5.7%) desired to have a doctor perform a physical exam. Critiques added as free text included lack of EMCs’ willingness to prescribe antibiotics or other medications (n=7, 4%), poor interaction with EMCs (n=2, 1.1%), and poorly defined billing/cost (n=1, 0.6%).

There was a total of 785 distinct chief complaints recorded in the time period of this study. This includes survey responders and nonresponders for a total of 3097 patients. Patients entered free-text complaints when registering for a VF visit. The most common chief complaints were upper respiratory infection (n=638, 21.2%), dermatologic complaints (n=192, 6.4%), ophthalmologic complaints (n=190, 6.3%), gastrointestinal complaints (n=186, 6.2%), and urinary complaints (n=166, 5.5%). A total of 306 of 3097 (10.2%) patient chief complaints were left blank (Table 5).

**Table 1:** Demographic data of the study population (N=176).

Characteristic		Patient, n (%)
**Age (years)**		
	0-4	3 (1.7)
	5-12	4 (2.3)
	13-18	5 (2.9)
	19-24	6 (3.5)
	25-25	21 (12.2)
	35-44	37 (21.3)
	45-54	42 (24.4)
	66-64	31 (18.0)
	65 or older	23 (13.4)
**Gender**		
	Male	44 (25.0)
	Female	130 (73.9)
	Gender-fluid	1 (0.6)
	Transgender male	0 (0.0)
	Transgender female	0 (0.0)
	Prefer not to answer	1 (0.6)
**Race**		
	White	150 (86.2)
	Black or African American	20 (11.5)
	Asian	2 (1.1)
	American Indian or Alaska Native	2 (1.1)
	Native Hawaiian or other Pacific Islander	0 (0.0)
**Ethnicity**		
	Hispanic	12 (6.8)
	Non-Hispanic	164 (93.2)

**Table 2:** Established and new patient data of study population (N=176).

Previously seen at Wake Forest Baptist Health	Patients, n (%)
<1 month ago	86 (48.1)
Within last 1 year	67 (38.1)
Over 1 year ago	15 (8.6)
Never	7 (4.0)

**Table 3:** Preferred location of care without access to Virtual First (N=176).

Alternative care	Patients, n (%)
Urgent care center	87 (49.4)
None	34 (19.3)
Primary care provider	28 (15.9)
Emergency department	26 (14.8)
Other (health department)	1 (0.6)

**Table 4:** Values and criticisms of Virtual First (N=176).

Responses		Patients, n (%)
**Valued aspects of care**		
	Care in comfort of home	137 (77.8)
	Availability of appointments	105 (59.6)
	Not waiting in a lobby	100 (56.8)
	Decreased infectious exposure	89 (50.6)
	Instant access to provider^a^	5 (2.8)
	Quality EMC^a,b^	4 (2.3)
	Did not value^a^	1 (0.6)
**Critiques of care**		
	Nothing^a^	58 (33.0)
	Poor reception or connectivity	47 (26.7)
	Inability to obtain labs or imaging	23 (13.1)
	Needed in-person medical care despite Virtual First visit	14 (13.1)
	Wanted a doctor to perform a physical exam	10 (5.7)
	EMC would not prescribe desired antibiotics or other medications^a^	7 (4.0)
	Bad interaction with EMC^a^	2 (1.1)
	Billing not well explained^a^	1 (0.6)

^a^Free-text response categories.

^b^EMC: emergency medicine clinician.

**Table 5:** Patients’ chief complaints (N=3097).

Chief complaints	Patients, n (%)
Upper respiratory infection	638 (21.2)
Dermatologic complaints	192 (6.4)
Ophthalmologic complaints	190 (6.3)
Gastrointestinal complaints	186 (6.2)
Urinary complaints	166 (5.5)
Left blank	306 (10.2)

## Discussion

### Principal Findings

This cross-sectional survey study of patients who used VF shows that access to an EMC via a virtual visit has the potential to reduce in-person visits to acute care facilities like UCCs and EDs. Of 177 patients, 113 (64.2%) would have gone to a UCC or ED for medical evaluation if VF had not been available to them. The goal of VF is to both reduce unnecessary visits to the ED and direct patients to the appropriate site of care if in-person care is needed. This initial data is promising as it reveals that patients chose VF to address complaints for which they would have otherwise sought at an acute care facility. The responses to the survey also demonstrated that most patients are not seeking care via VF to address concerns for which they would regularly see their PCP. VF is not designed to be a replacement for building a relationship with a PCP or managing chronic illnesses, so it is encouraging that most survey respondents accessed VF for complaints they believed could not be handled in the outpatient office setting. This study also revealed that many patients are satisfied with the care received during a VF visit. Factors of convenience such as care from the comfort of home, availability of appointments, and not waiting in a lobby were the most highly selected responses for the most valued aspect of care. Regarding the critiques of VF, the most common concern was connectivity. EMCs call patients to complete the visit if technical difficulties arise. Lack of video or poor-quality video during a visit can significantly impact an EMCs ability to assess patients. Continued work troubleshooting technical issues such as video quality and connectivity will be important to ensure quality care is provided virtually. Additional research will be needed to determine that connectivity is not related to internet or cell phone carriers or zip codes to ensure more universal accessibility to VF. Patients also selected “inability to obtain labs or imaging” as a critique of VF. During the study period, VF was able to increase the number of tests that could be obtained after the virtual visit. This may have reduced the number of patients that selected this option later in the study period. Lastly, the second-most common free-text critique was regarding EMCs’ refusal to prescribe antibiotics or other medications that the patient felt were necessary. While this can be a source of frustration during in-person assessments as well, VF providers do not have access to real-time testing like urinalysis or streptococcal screens to provide additional data to patients regarding a nonbacterial source of their complaint. Virtual visits have unique challenges and require EMCs to gain rapport quickly and manage expectations regarding the visit early. Collection of this patient feedback will allow VF to address deficiencies in the virtual model, as evidenced by the evolving ability to obtain outpatient testing. EMCs are uniquely equipped for acute care virtual visits. VF’s innovative employment of EMCs allows for a patient’s acute care needs to be treated virtually if feasible. If comprehensive management of the patient’s complaint cannot be performed virtually, EMCs understand the capabilities of local EDs, the feasibility of subspecialty consultations, and the available testing and imaging within the ED to better direct patients to the appropriate site for their acute care needs. This has the potential to substantially decrease patient costs because patients are given the appropriate destination for in-person care, reducing the likelihood of the need for transfer and multiple ED visits. For patients with acute care needs that can be evaluated and treated virtually without referral to an in-person acute care facility, the charge for a VF visit ranges from US $100 to US $700. This is a significant cost saving to the patient when compared to US $4969, the average charge for patients treated and discharged from US EDs in 2019 [19].

### Comparison to the Current Literature

The current literature shows that the diagnoses most commonly seen by virtual acute care services include sinusitis, upper respiratory infection, urinary tract infection, conjunctivitis, and dermatologic conditions [20,21]. In our study, specific patient diagnoses were not linked to each survey, but the most common chief complaints from patients were reflective of what is seen in the literature except for an increased predominance of gastrointestinal complaints in our patient population.

Additionally, this postvisit survey supports the claim of previous literature [1-4] that patients have a high level of satisfaction with telemedicine. Upon review of survey responses, especially free-text responses, patients appreciate the convenience of accessing the health care system virtually with EMCs. It is also important to point out that when asked to provide critiques of VF, one-third of survey responses did not select a predefined category but instead wrote “Nothing.” This appreciation for the conveniences of accessing the health care system for acute care virtually lends itself toward the ability of this telemedicine model to extend beyond the COVID-19 pandemic.

Lastly, like previous studies of demographic trends in telemedicine use, our sample population was primarily white and female [22]. The patient population served by VF has been outlined in Table 1. Other studies have also noted disparities in telemedicine use by patients with private insurance and higher incomes, and those living in urban areas [23-25].

### Limitations

The aim of this study is to understand how the option of virtual video visits changes how patients seek initial care for acute care needs. First, the survey created for this study did not undergo validation or reliability testing. As highlighted by Bull et al [26], validity testing in patient-reported experience surveys can be challenging as there is often not a comparable or gold standard survey for the exact patient experience being studied. Survey reliability is also inherently challenging in the patient-reported experience survey as each survey looks at a unique, one-time event. Second, information was not collected to link a patient survey to the patient’s VF encounter. Because of this, the ultimate disposition after the patient’s VF encounter is unknown. Third, a patient with multiple VF visits received a survey invite for each visit and could complete the VF survey multiple times. This has the potential to result in multiple similar entries that could skew the results toward a patient’s preference for seeking care in a specific setting and influence overall patient satisfaction, as repeat patients are more likely to have been satisfied with their care. Fourth, the response rate to the survey was low, which can raise concerns about nonresponse bias and generalizability. Postvisit surveys continue to be collected from patients that access VF to gather additional insight into patient preferences and actions to increase our overall response numbers. Press Gainey, a similar web-based survey, has an average response rate of 13.5% [27]. Responders were found to be different than nonresponders as they were more frequently older, white, employed, college educated, and married. We were unable to track the number of postvisit surveys that were sent, and while EMCs were encouraged to send the link after every visit, it is possible that the response rate is higher because the number of surveys sent does not equal the number of patients seen. Lastly, this is a cross-sectional survey study and is therefore limited in its ability to derive causation as well as association. This data alone does not have the ability to determine if VF visits directly result in decreased in-person visits to acute care facilities. It is, however, useful for monitoring how patients are using this resource and their evaluation of this resource over a specific period of time.

### Future Directions

This study evaluates the question of where a patient would seek care if VF had not been available to them. It does not, however, identify if patients who sought care at VF were referred to or sought additional care in person. Further research will be needed to explore whether VF successfully decreases unnecessary ED visits. This will be best accomplished by both tracking which patients are referred to a UCC or ED as well as those that are deciding to seek a second opinion in person after a VF visit. Additionally, for those that stated they would have sought care at their PCP’s office, further studies investigating the lack of appointment availability or other factors are necessary. Lastly, the current literature, in addition to trends seen in our data, implores our health care system to intentionally provide education about this program to minority, lower income, and uninsured patients as well as research underlying disparities that may be inherent to telemedicine. In addition to the desire to improve access to acute care, VF also hopes to expand the use of the platform to patients that have not accessed our health care system in the past. VF was primarily marketed to existing patients of our health care system; however, 7 of 176 (4%) VF patients were new to the system. Curiously, 34 of 176 (19.3%) patients who accessed VF would not have sought care for their acute complaint if VF were not available to them. The exact patient complaints for these encounters are not known as the survey responses are not linked directly to the virtual visit. This is a subgroup of patients important to understand in the future as they could represent patients who are underserved or lack the ability to engage with health providers in person. Further efforts should be made to market this new way to access the health care system, especially in areas of the state that have fewer health care resources and underserved populations, and are geographically distant from high-resource tertiary centers.

### Conclusions

VF has the potential to restructure how patients seek medical care by connecting EMCs with patients prior to ED arrival. Without the option of VF, 64.2% (113/177) of patients would have sought care at an acute care facility. We attribute the successful reduction in UCC and ED visits to EMCs being involved in the management of these visits.

## Abbreviations

ED: emergency department
EMC: emergency medicine clinician
PCP: primary care provider
UCC: urgent care center
VF: Virtual First

## Data Availability

The data sets generated or analyzed during this study are available from the corresponding author upon reasonable request.
